# Sinus‐like dilatations of the mammary milk ducts, Ki67 expression, and CD3‐positive T lymphocyte infiltration, in the mammary gland of wild European rabbits during pregnancy and lactation

**DOI:** 10.1111/joa.12824

**Published:** 2018-05-07

**Authors:** Katherine Hughes, Christine J. Watson

**Affiliations:** ^1^ Department of Veterinary Medicine University of Cambridge Cambridge UK; ^2^ Department of Pathology University of Cambridge Cambridge UK

**Keywords:** lactation, mammary gland, mastitis, milk duct, pregnancy, rabbit, sinus, tumour

## Abstract

Sinus‐like dilatations of the mammary duct are recognisable in the mammary gland of pregnant and lactating wild European rabbits. These dilatations exhibit a bilaminar epithelial lining, with luminal epithelial cells expressing basal and lateral E‐cadherin. Occasional binucleated mammary epithelial cells are present in the luminal layer. Underlying the luminal epithelial cells is a basal layer of cytokeratin 14‐positive cells, supported by a thin layer of fibrous tissue. Multi‐segmental epithelial proliferation, as indicated by Ki67 expression, is apparent in the luminal epithelial cells, suggesting a capacity for division during pregnancy and lactation. CD3‐positive T lymphocytes are present both intraepithelially, suggesting exocytosis, and in foci subjacent to the ductular epithelium. We consider that sinus‐like dilatations of the mammary duct may have the potential to give rise to a subset of the mammary gland neoplasms classified as ductal in origin. Milk accumulation in these sinus‐like dilatations is likely to provide a niche for bacterial replication in cases of mastitis in rabbits. These structures are an important component of the innate immune system of the mammary gland, both as a physical barrier and as an interface between the milk and mammary immune cells.

## Introduction

Over the last 25 years, diverse sources have indicated that rabbits are an increasingly popular pet species in many parts of the world (Grant et al. 2017). From 1994 to 2003 rabbits were the most frequently presented species at the Clinic of Zoo Animals, Exotic Pets and Wildlife at the University of Zurich, with the number of rabbits increasing significantly during that time (Langenecker et al. 2009). In the USA, the number of pet rabbits increased between 2001 and 2006 (Shepherd, 2008). In the UK in 2017 an estimated 2% of the UK population owned a pet rabbit, with the pet rabbit population estimated at 1.1 million (People's Dispensary for Sick Animals, 2017). In Andalusia, Spain, where traditionally rabbits are considered an important meat‐producing species, it has been suggested that young people's perceptions of the rabbit may be shifting from viewing it as a livestock species to a pet (González‐Redondo & Contreras‐Chacón, 2012). Whereas rabbits are growing in prominence as pets, in many countries they instead, or in addition, constitute an important farm animal species (Kylie et al. 2017).

Different types of mammary gland pathology are an important consideration in both pet and commercial rabbits. Pet rabbits, especially those which are not neutered, may develop mammary tumours (Van Zeeland, 2017) and a subset of these tumours are classified as ductal in origin (Schöniger et al. 2014; Baum & Hewicker‐Trautwein, 2015). In meat‐producing rabbits, mastitis affecting the breeding females may be a major issue in some systems (Adlam et al. 1976; Segura et al. 2007; Corpa et al. 2009; Rosell & De La Fuente, 2009; Viana et al. 2011; Guerrero et al. 2015) and mastitis is also an occasional problem in laboratory rabbits (Bergdall & Dysko, 1994). To address and understand fully the range of mammary gland pathology recognised in pet rabbits and those kept for meat production, a comprehensive and species‐specific understanding of mammary gland biology in the rabbit is desirable.

Rabbits have four or five pairs of mammae (Lossi et al. 2016) and approximately six ductal systems per mamma (Cowie, 1974), in contrast to mice, cattle, sheep, and goats, which have a single ductal system per gland (Rowson et al. 2012). In this respect, the rabbit mammary gland more closely resembles the human breast, which has 4–18 ductal systems (Cowie, 1974; Love & Barsky, 2004; Ramsay et al. 2005). This notable variation in the number of ductal systems, or galactophores, per mamma is due to developmental differences between species. The rabbit is considered a ‘multi‐sprouting species’ (Oftedal & Dhouailly, 2013) because at embryonic day 26, division of the mammary bud results in sprouts which each give rise to a primary milk canal. Consequently, in the rabbit and human, the mamma is divided into different ductal systems or sectors by the formation of multiple mammary trees, each with a primary milk canal (Macias & Hinck, 2012; Propper et al. 2013). In the rabbit it has been proposed that the secretory alveoli of the gland constitute the primary site of milk storage (Calvert et al. 1985) and it has been suggested that the rabbit mammary gland does not exhibit lactiferous sinuses (Cowie, 1974).

The purpose of this study was further to investigate aspects of the histology of the mammary gland of wild European rabbits, *Oryctolagus cuniculus*, during pregnancy and lactation. In particular, we wished to explore the possibility of the presence of sinus‐like dilatations of the milk ducts. Our objectives were to assess proliferation within the glandular epithelial cells, and infiltration of the ducts by CD3‐positive T lymphocytes, to better characterise the sinus‐like dilatations.

## Methods

### Animals

Rabbits that had been shot for population management were donated for research and teaching purposes to the veterinary anatomical pathology service of the Department of Veterinary Medicine, University of Cambridge. Rabbit cadavers were stored at 4 °C within 3 h of being shot. For each cadaver in the study, a macroscopic postmortem examination was conducted by a single board‐certified veterinary pathologist. Postmortem examinations, including assessment of the mammary glands and reproductive tract, were carried out within an estimated 72 h of death. Cadavers were either in stage one or two of the five‐stage scale of postmortem change (Brooks, 2016). Where appropriate, stage of pregnancy was determined by assessment of fetal crown‐rump measurement and/or fetal weight (Hagen, 1974).

### Histology

Mammary tissue from seven pregnant and five lactating rabbits was collected in 10% neutral‐buffered formalin, and was subsequently processed and sectioned at 5 μm. For assessment of the number of mammary ducts, a randomly selected mamma was chosen from each of 10 rabbits and transverse sections through the teat were cut at 1 and 2.5 mm (fixed tissue measurements) from the distal aspect of the teat. Only transverse sections were analysed; tangential sections were not included in further analyses. For other analyses, a second randomly chosen mamma was selected and parasagittal sections were cut through the teat and underlying mammary tissue. Staining with haematoxylin and eosin (H&E) and Masson's trichrome followed standard histological protocols.

### Immunohistochemistry

Tissue sections exhibiting evidence of postmortem autolysis were excluded from immunohistochemical (IHC) staining. IHC staining for E‐cadherin (dilution 1 : 200; mouse monoclonal, clone NCH‐38, catalogue number M3612, Dako Pathology/Agilent Technologies LDA UK, Cheadle, Cheshire, UK), cytokeratin 14 (dilution 1 : 200; mouse monoclonal, catalogue number Ab7800, Abcam, 330 Cambridge Science Park, Cambridge, Cambridgeshire, UK), Ki67 (dilution 1:100; mouse monoclonal, clone MIB‐1, catalogue number M7240, Dako Pathology/Agilent Technologies LDA UK), and CD3 (dilution 1 : 150; mouse monoclonal, clone F7.2.38, catalogue number M7254, Dako Pathology/Agilent Technologies LDA UK) followed a routine protocol using an automated IHC system (Dako Pathology/Agilent Technologies). Negative control slides were prepared using species‐matched immunoglobulins.

### Digital slide scanning

Selected sections stained with H&E and Masson's trichrome were scanned at 40× (Nanozoomer 2.0RS, C10730, Hamamatsu Photonics, Hamamatsu City, Japan). Viewing software (NDP.view2, Hamamatsu Photonics) was utilised to analyse these sections and to capture images.

## Results

### The apparent number of mammary ducts varies between rabbits

**Figure 1:**
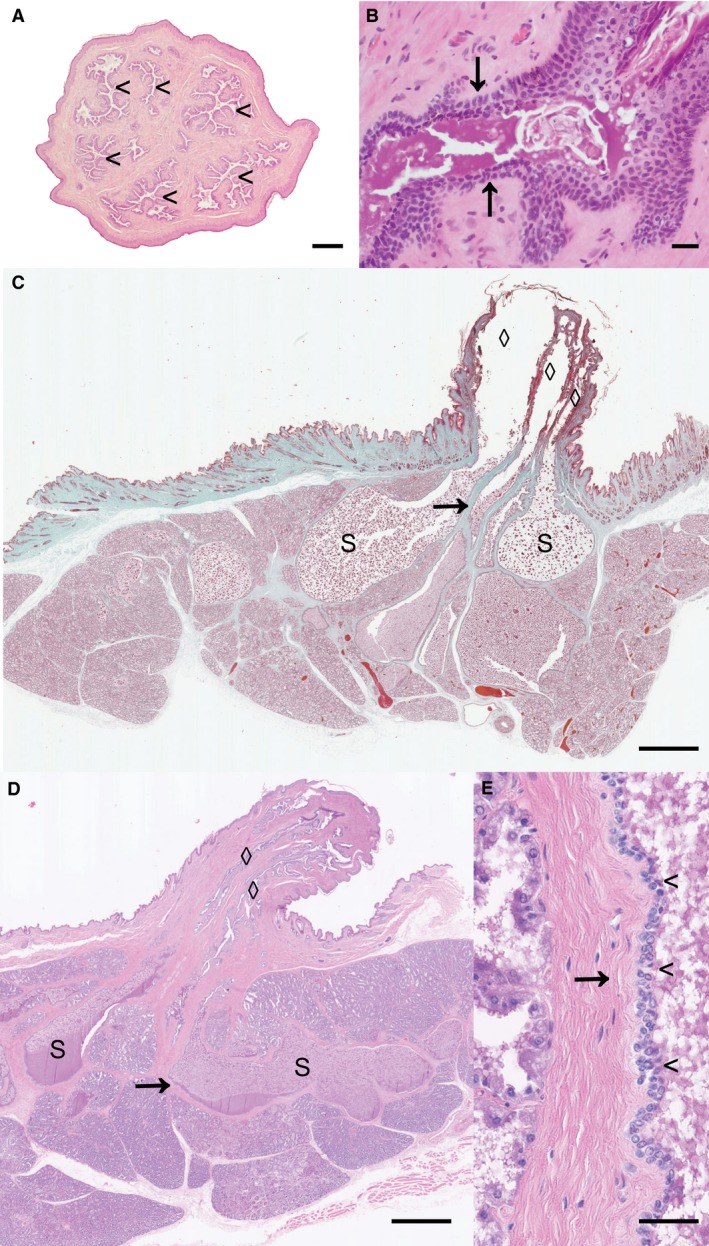
Sinus‐like dilatations of the mammary ducts of the rabbit mammary gland. (A) Transverse section approximately 2 mm from the distal aspect of the teat of a lactating rabbit. Six major ducts are apparent (arrowheads). (B) Parasagittal section through the teat canal‐mammary duct junction of a pregnant wild rabbit, estimated 22 days gestation. Arrows indicate transition from a stratified squamous to a bilaminar ductular epithelium. (C,D,E) Parasagittal sections through the teat and mammary tissue of a pregnant wild rabbit, estimated 27 days gestation (C) and a lactating rabbit (D,E). Multiple ducts (diamonds) exhibit sinus‐like dilatations (s), which are supported by thin layers of fibrous tissue (arrows). (E) Parasagittal section of a sinus‐like dilatation demonstrating the double layered epithelial lining (arrowheads). The lumen is to the right of the photomicrograph and the sinus‐like dilation is supported by fibrous tissue (arrow). Scale bars: 500 μm (A), 50 μm (B), 1.5 mm (C), 1 mm (D) and 40 μm (E). H&E stain (A,B,D,E); Massons trichrome stain (C). Images are representative of 10 (A) to 12 (B–E) biological repeats.

Transverse sections from the teat of 10 rabbits (five pregnant, five lactating) were analysed. The mean ± SD and median number of mammary duct cross‐sections per teat were 6.7 ± 0.76 and 7, respectively, which was similar to previous reports suggesting that the rabbit commonly has six galactophores (Cowie, 1974) (Fig. 1A). Interestingly, frequent small diameter ducts were observed in cross‐section, and we interpreted these as most likely resulting from secondary spouting (Oftedal & Dhouailly, 2013). Although the presence of adjacent small diameter ducts made counting somewhat subjective, where transverse sections taken at 1  and 2.5 mm were both available for analysis from the same rabbit, the same number of ducts were counted in both cases, increasing confidence in the results (data not shown).

### The mammary ducts exhibit an abrupt transition from stratified squamous epithelium to bilaminar epithelium at the junction with the teat canal

When analysing sagittal sections, we observed an abrupt transition from a stratified squamous epithelium of the teat canal to a bilaminar ductular epithelium lining the mammary duct (Fig. 1B) similar to the abrupt transition from stratified squamous epithelium to double‐layered epithelium between the teat canal and teat cistern described in ruminants (Nickerson & Akers, 2011).

### Sinus‐like dilatations of the mammary milk duct are present in the rabbit during pregnancy and lactation

Analysis of parasagittal sections of mammae from 12 rabbits (seven pregnant, five lactating) revealed the presence of sinus‐like dilatations of the milk ducts during pregnancy and lactation (Fig. 1C,D). In all 12 animals, we identified pronounced dilatations of the milk ducts that measured up to 2.97 mm (fixed tissue) in diameter (Fig. 1C) and 4.22 mm (fixed tissue) in length (Fig. 1D). The sinus‐like dilatations were lined by a bilaminar epithelium (Fig. 1E), continuous with the milk duct epithelium, and were supported by a thin layer of collagenous connective tissue. Although no striking morphological differences were detected between pregnant and lactating rabbits, we subjectively considered that the dilatations were larger in those rabbits that were in late gestation or lactation.

**Figure 2:**
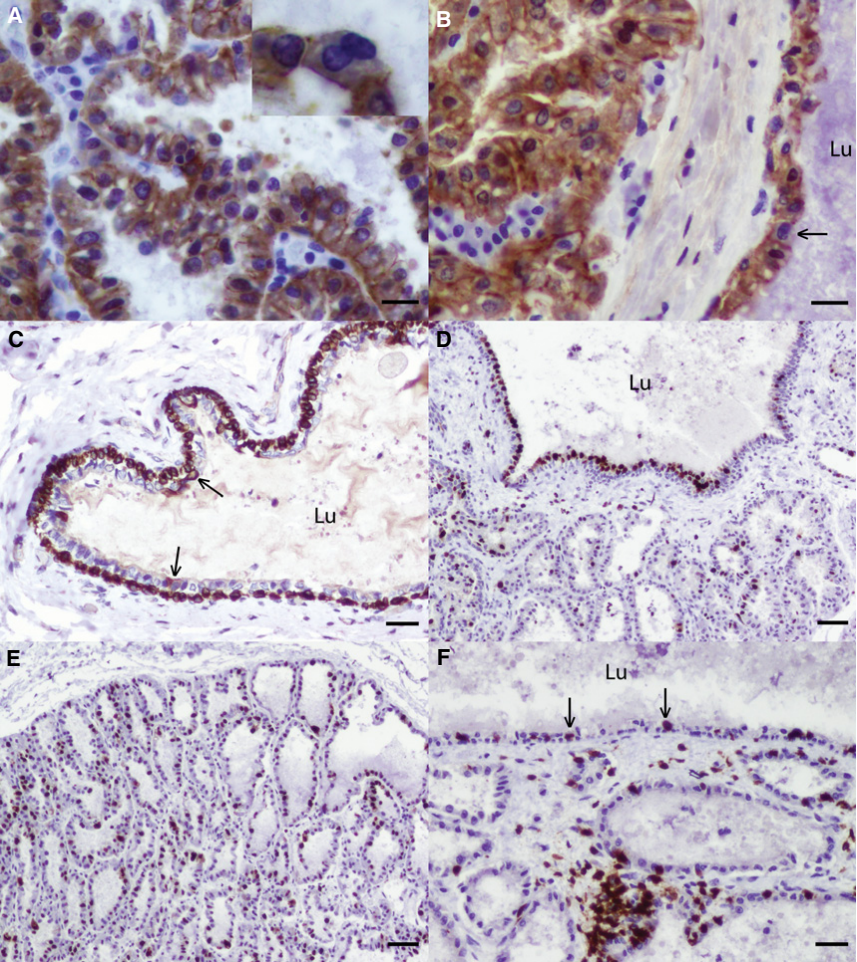
The rabbit mammary gland during pregnancy and lactation. (A,B) Immunohistochemical staining for E‐cadherin demonstrates expression at the basal and lateral aspects of luminal mammary epithelial cells forming mammary alveoli and lining the sinus‐like dilatations of the mammary duct. Mammary gland from two different lactating rabbits. Inset shows an apparently binucleated mammary alveolar luminal epithelial cell. Arrow indicates an apparently binucleated epithelial cell lining a sinus‐like dilatation of the mammary duct. (C) Immunohistochemical staining for cytokeratin 14 demonstrates intense cytoplasmic expression in the basal epithelial layer of the sinus‐like dilatations, and rare cytokeratin 14‐positive luminal cells (arrows). Pregnant rabbit, estimated 17–18 days gestation. (D,E) Immunohistochemical staining for Ki67 demonstrates multi‐segmental expression in the luminal epithelial cells lining the sinus‐like dilatations of the duct and in mammary alveolar luminal epithelial cells. Lactating rabbit. (F) Immunohistochemical staining for CD3 demonstrates CD3‐positive T lymphocytes both in clusters between adjacent mammary alveoli, subjacent to the sinus‐like structures, and in intraepithelial foci (arrows). Lactating rabbit. Scale bars: 30 μm (A,B), 50 μm (C,F) and 100 μm (D,E). Haematoxylin counterstain; Lu: lumen of sinus‐like structure. Images are representative of three biological repeats.

E‐cadherin is important in the adherence of luminal epithelial cells to neighbouring cells (Cowin et al. 2005) and in the present study, cross‐reactivity of the antibody to rabbit E‐cadherin was confirmed by detection of E‐cadherin immunolabelling particularly at the basolateral aspects of luminal mammary alveolar epithelial cells (Fig. 2A), similar to the pattern we and others have observed and described in the mouse mammary gland (Alford & Taylor‐Papadimitriou, 1996; Cowin et al. 2005; Hughes et al. 2012, 2016). In spite of a mild level of postmortem autolysis, we observed similar lateral and basal staining in the luminal epithelial cells lining the sinus‐like dilatations (Fig. 2B). Additionally, E‐cadherin staining allowed us to delineate the cell boundaries of adjacent luminal epithelial cells such that we were able to appreciate occasional binucleated luminal epithelial cells in alveoli and in the lining of the sinus‐like dilatations (Fig. 2A,B).

Cytokeratin 14 is an intermediate filament expressed by myoepithelial cells which has been used to identify mammary epithelial basal cells, myoepithelial cells or basal‐like carcinomas in numerous studies involving tissues from a number of different species, including the cow (Alkafafy et al. 2012), dog (Matos et al. 2006), and horse (Hughes et al. 2015). We assessed expression of cytokeratin 14 in the mammary ducts, and particularly in the sinus‐like dilatations. Cross‐reactivity of the antibody was confirmed by detection of cytokeratin 14 expression in the basal layers of the epidermis of the teat (data not shown) (Wei et al. 2016). Intense cytoplasmic expression of cytokeratin 14 was detected in the basal layer of the mammary duct and the sinus‐like dilatations of the duct but not in the majority of epithelial cells making up the luminal layer, although rare individual luminal cells strongly expressed cytokeratin 14 (Fig. 2C).

### A proportion of mammary ductal epithelial cells exhibit Ki67 expression during pregnancy and lactation

Given that Ki67 expression, used to delineate the proportion of a cell population which is actively cycling, has been used to demonstrate proliferation of mammary epithelial cells during pregnancy in both human (Suzuki et al. 2000) and mouse (Oliver et al. 2012), we wished to analyse Ki67 expression in the mammary epithelial cells of the sinus‐like dilatations of the milk duct to assess whether there was extensive cell proliferation within the lining epithelium at these developmental time points.

Ki67 expression within the skin overlying the gland indicated specificity of cross‐reactivity of the antibody (data not shown). Interestingly, there was strikingly multi‐segmental expression of Ki67 in the luminal epithelial cells lining the sinus‐like dilatations of one lactating rabbit, suggesting that the ductular epithelial cells may undergo proliferation during the postnatal mammary developmental cycle (Fig. 2D). Ki67 expression was also multifocally abundant in alveolar epithelial cells during both pregnancy and lactation (Fig. 2E).

### CD3‐positive T lymphocytes multifocally infiltrate the sinus‐like dilatations of the mammary duct

As lymphocytes have been described as the predominant immune cell in the teat cistern of ewes (Mavrogianni et al. 2007), we hypothesised that CD3‐positive T lymphocytes might be found in association with the sinus‐like dilatations of the mammary duct which we had observed in pregnant and lactating rabbits. Immunohistochemical staining revealed that CD3‐positive T lymphocytes were present multifocally, both in intraepithelial foci (exocytosis) and subjacent to the epithelium of the ductular dilatations. CD3‐positive T lymphocytes were also irregularly interspersed between mammary alveoli, but there was no apparent pattern to their distribution (Fig. 2F).

## Discussion

In this study we have corroborated previous reports suggesting that the rabbit commonly has multiple galactophores (Cowie, 1974), although in our sample population the median number of galactophores per rabbit was seven rather than six. However, it is important to acknowledge the limitations of assessment of galactophores by histological analysis. The human literature underlines the benefit of ultrasonographic assessment of the anatomy of the lactating breast (Ramsay et al. 2005) and we highlight in this study that identification of patent galactophores vs. smaller diameter ducts is subjective and open to interpretation. The human breast also has multiple ductal systems (Cowie, 1974; Going & Moffat, 2004; Love & Barsky, 2004; Ramsay et al. 2005; Koyama et al. 2013) and, in this respect, the rabbit could therefore be considered a better model of the human breast compared with the mouse.

Importantly, in this report we have identified sinus‐like dilatations of the mammary ducts in pregnant and lactating wild European rabbits. Similar to the rest of the duct, these dilatations exhibit a bilaminar epithelial lining, with luminal epithelial cells expressing basal and lateral E‐cadherin. There is a basal layer of cytokeratin 14‐positive cells, supported by a thin layer of fibrous tissue. We suggest that these sinus‐like dilatations of the mammary duct may have the potential to give rise to a subset of the rabbit mammary gland neoplasms classified as ductal in origin (Schöniger et al. 2014; Baum & Hewicker‐Trautwein, 2015).

Multi‐segmental proliferation, as indicated by Ki67 expression, is apparent in the luminal epithelial layer. Although it is now suggested that mammary ducts in the human breast predominantly function as milk conduits rather than storage sites (Ramsay et al. 2005), earlier descriptions of infant sucking suggested that milk ducts near the nipple acted as a ‘reservoir’ for milk (Woolridge, 1986). Our histological observations of the rabbit mammary gland during pregnancy and lactation appear to support a similar hypothesis, with dilatations of the duct subjacent to the teat.

Although dilatations of the milk duct are clearly apparent in histological sections, conventional histological assessment has limitations, as it provides a two‐dimensional representation of a three‐dimensional, dynamic structure. In the study of the human breast, clinical imaging modalities such as ultrasound (Ramsay et al. 2005) have provided further insights. However, in veterinary species, welfare considerations are paramount, and this approach may be prohibited because of the use of sedation in pregnant and lactating rabbits. An alternative methodology which would likely yield informative results is high‐resolution three‐dimensional imaging using tissue clearing, which has recently been described in the mouse mammary gland (Davis et al. 2016; Lloyd‐Lewis et al. 2016). A potential strength of our report is that the rabbits were wild and so no impact of artificial husbandry in a farm or laboratory environment is likely. Conversely, a limitation of our study is that we have no information regarding lactational history in these animals – for example, we do not know the parity of the rabbits, we cannot rule out the possibility of concurrent pregnancy and lactation, and we also do not know when the rabbits last nursed their young.

We report the presence of suspected binucleated epithelial cells within both the rabbit mammary alveoli and the luminal layer of the ductular dilatations. Binucleated mammary epithelial cells have been identified during lactation in the mouse, human, seal, wallaby, and cow (Banerjee et al. 1971; Banerjee & Wagner, 1972; Kriesten, 1984; Oliver et al. 2012; Rios et al. 2016; Smith, 2016). Interestingly, we noted the presence of binucleated cells both within the luminal epithelial cell population of the mammary alveoli and also rarely in the ductular dilatations. Three‐dimensional imaging combined with immunofluorescence staining for E‐cadherin would facilitate accurate quantification of the proportion of binucleated mammary epithelial cells within the rabbit mammary gland during pregnancy and lactation (Davis et al. 2016; Lloyd‐Lewis et al. 2016; Rios et al. 2016).

Strikingly, we also noted that within the bilaminar epithelium lining the sinus‐like dilatations, rare individual, luminal cells strongly expressed cytokeratin 14. Although this finding was unexpected, others have reported that in mice embryonically derived, long label‐retaining cells are present within the primary mammary ducts near the nipple region, and that the majority of these cells are positive for cytokeratin 14 (Boras‐Granic et al. 2014). It is therefore possible that the rare luminal cytokeratin 14‐positive cells we observed represent a bipotent progenitor population, although this suggestion is entirely speculative based on the data available.

CD3‐positive T lymphocytes are present in multifocal locations both adjacent to the sinus‐like dilatations of the mammary duct and infiltrating the epithelial lining. This is similar to previous reports in the cow, in which lymphocytes were described as the most common infiltrating cell in the teat cistern (Nickerson & Pankey, 1983). Intraepithelial lymphocytes are suggestive of exocytosis. Migration of leucocytes into the milk from the blood is a mammary defence mechanism described in other species such as the cow (Nickerson, 1985) and we suggest that the presence of intraepithelial CD3‐positive lymphocytes in the epithelial lining of these sinus‐like dilatations in the rabbit likely fulfil a similar function.

Given that milk appears to accumulate in the sinus‐like dilatations we describe, we speculate that the presence of milk could provide a nidus for bacterial replication in cases of mastitis in rabbits. Mastitis is known to be a particular welfare problem in commercial rabbitries, with this disease listed as the second most common cause for culling does in one study (Rosell & De La Fuente, 2009) and the reason for culling 33.3% of culled does in an separate investigation (Segura et al. 2007), with both of these analyses focusing on Spanish commercial rabbit farms. A further study analysing data from rabbitries in Spain and Portugal indicated a mastitis prevalence of 4% (Sánchez et al. 2012). Bacterial pathogens could potentially track into the gland through the teat canal, as in ruminants (Nickerson, 1985), and from here may proliferate in milk that is present in the sinus‐like structures. We therefore hypothesise that these structures may be an important component of the innate immune system of the mammary gland, both as a physical barrier and as an interface between the milk and mammary immune cells, and furthermore could potentially favour or facilitate bacterial spread and replication once the teat canal has been breached. Although this study has utilised tissue from wild rabbits, we consider that our findings are likely applicable to pregnant and lactating does being bred as part of a laboratory colony, as pets or on a commercial rabbit farm.

## Author Contributions

K.H. conceived and designed the study, acquired, analysed and interpreted data, and wrote the manuscript. C.J.W. contributed to data interpretation and commented on the manuscript. Both authors have approved the submitted article.
